# Doll therapy in residential dementia care in China: protocol for an exploratory pilot and feasibility mixed-methods cluster randomized controlled trial

**DOI:** 10.3389/fpubh.2026.1842340

**Published:** 2026-07-02

**Authors:** Zhenti Cui, Rongrong Guan, Tianrui Xue, Yongchang Sun, Maw Pin Tan, Mas Ayu Said, Yingdong Cao

**Affiliations:** 1 School of Medicine, Sias University, Zhengzhou, Henan, China; 2 Department of Social and Preventive Medicine, Universiti Malaya, Kuala Lumpur, Malaysia; 3 School of Art and Design, Zhengzhou University of Industrial Technology, Zhengzhou, Henan, China; 4 Division of Geriatric Medicine, Department of Medicine, Faculty of Medicine, Universiti Malaya, Kuala Lumpur, Malaysia

**Keywords:** dementia, doll therapy, cluster randomized controlled trial, nursing homes, psychotropic medications, activities of daily living, China

## Abstract

**Background:**

Behavioral and psychological symptoms of dementia commonly drive psychotropic prescribing in residential care. Doll therapy is a person-centered, attachment-informed intervention that may reduce distress and support engagement, but evidence from Chinese nursing homes remains limited. This protocol describes an exploratory pilot and feasibility mixed-methods cluster randomized controlled trial of a staff-mediated doll-therapy care package plus routine nursing care versus routine nursing care alone.

**Methods and analysis:**

Phase I is a pragmatic, parallel-group, 1:1 cluster randomized pilot trial in six nursing homes in Zhengzhou, China, targeting 142 residents with dementia. Intervention homes will receive a structured doll-therapy introduction of eight planned sessions over approximately 15 days, continued access to a personal doll during routine care, staff training, structured interaction, relational engagement, and routine-care implementation; control homes will provide routine nursing care alone. Assessments occur at baseline, 6 weeks, and 12 weeks. The registered primary outcome is class-specific mean daily medication dose over 12 weeks for antipsychotics, anxiolytics, and antidepressants, interpreted as an indirect medication-stewardship signal. The main proximal clinical outcome is total Neuropsychiatric Inventory-12 score. Secondary and contextual outcomes include Barthel Index, cognitive enhancer use, traditional Chinese medicine use, and structured engagement with the doll. Phase II uses semi-structured interviews with staff and family caregivers from intervention homes to examine acceptability, implementation, training needs, cultural fit, and ethical considerations. Quantitative analyses will emphasize effect estimates, confidence intervals, cluster-level summaries, and randomization/permutation-based robustness checks; qualitative data will be analyzed thematically.

**Discussion:**

The study will provide feasibility, acceptability, fidelity, safety, preliminary clinical-signal, and implementation evidence to inform a future adequately powered cluster trial in Chinese residential dementia care.

**Trial registration:**

https://clinicaltrials.gov/study/NCT06506487, ClinicalTrials.gov, NCT06506487. Registered July 16, 2024.

## Introduction

Dementia is a major public health challenge associated with population ageing. It is characterized by progressive decline in cognitive function and loss of independence in everyday life, and it is commonly accompanied by behavioral and psychological symptoms such as agitation, anxiety, apathy, depression, wandering, and psychotic phenomena (1, 2). In residential care settings, these symptoms often drive caregiver distress, increase supervision needs, and lead to pharmacological escalation (3, 4). China carries a particularly large dementia burden because of rapid demographic ageing, increasing need for long-term care, and uneven access to diagnostic and care resources across regions (5).

Managing behavioral and psychological symptoms of dementia in nursing homes remains difficult. Antipsychotics, anxiolytics, and antidepressants are frequently used when residents show agitation, aggression, anxiety, or mood disturbance, yet these medications may increase sedation, falls, extrapyramidal symptoms, cerebrovascular events, pneumonia, or mortality risk in frail older adults (4, 6, 7). Non-pharmacological and person-centered interventions are therefore increasingly recommended as a core component of dementia care rather than as an optional add-on (8, 9). However, reductions in psychotropic medication use are not determined by behavioral improvement alone. Prescribing decisions in nursing homes may also be shaped by physician practice, medication-review routines, family preferences, staffing capacity, acute illness, and institutional policies. For this reason, medication-related outcomes in pragmatic psychosocial trials should be interpreted as indirect clinical and service-delivery indicators rather than as simple biological markers of intervention-specific effect.

Doll therapy is one such intervention. In doll therapy, a person living with dementia is offered a lifelike doll and may interact with it by holding, comforting, grooming, or talking to it. Existing reviews and small controlled studies suggest that doll therapy may reduce agitation and support emotional regulation, social interaction, and purposeful activity, but the evidence base remains limited by small samples, variable intervention protocols, inconsistent outcome selection, and incomplete reporting of implementation fidelity and ethical safeguards (10–12). Reported effects appear most consistent for agitation and observable mood-related domains, less consistent for daily functioning, and largely absent for medication-related outcomes such as psychotropic prescribing. Evidence from Chinese nursing homes remains especially limited, with few studies reporting standardized intervention protocols, fidelity monitoring, or medication-stewardship outcomes.

The conceptual rationale for the intervention is informed primarily by attachment theory. Dementia-related losses in memory, orientation, and social familiarity may intensify needs for security, soothing, and emotional regulation. Within this framework, a doll may function as a symbolic attachment object and support caregiving identity, comfort, and meaningful engagement for some residents (13, 14). The hypothesized causal chain from doll therapy to medication-related outcomes therefore involves two distinct steps: (i) a behavioral step in which attachment-informed engagement reduces dementia-related distress behaviors as captured by validated instruments such as the Neuropsychiatric Inventory-12 (NPI-12), and (ii) a prescriber response step in which clinicians and medication-review processes translate behavioral stabilization into reduced or stable psychotropic prescribing. The current pilot protocol does not directly measure attachment processes or formally test mediation; attachment-related mechanisms are inferred theoretically, while engagement records and qualitative interviews provide only indirect contextual evidence. Because the second step depends on prescriber behavior and institutional policy rather than on the intervention itself, any medication-related change observed in this trial will be interpreted alongside more proximal outcomes, particularly NPI-12, Barthel Index, and structured engagement records. In the Chinese care context, this rationale also needs to be considered alongside local cultural understandings of ageing, family caregiving, dignity, institutional care, and the coexistence of biomedical treatment and traditional Chinese medicine in routine dementia care (5, 15).

Doll therapy also remains ethically and culturally sensitive. Concerns have been raised about infantilization, deception, over-attachment, and respect for autonomy and dignity, especially if the intervention is imposed, poorly explained, or treated as a substitute for relational care (16, 17). Acceptability may vary according to residents’ prior life experiences, family expectations, staff attitudes, gendered caregiving histories, cultural beliefs about dolls and old age, and degree of cognitive impairment. These issues are central to the present protocol rather than peripheral implementation details.

Accordingly, the present study uses an exploratory pilot and feasibility mixed-methods design to assess the staff-mediated doll-therapy care package in Chinese residential dementia care. The quantitative phase compares the staff-mediated doll-therapy care package plus routine nursing care with routine nursing care alone on registered medication-related outcomes, NPI-12, Barthel Index, cognitive enhancer use, traditional Chinese medicine use, and participant engagement with the doll over 12 weeks. Because the number of randomized clusters is small, the trial is intended to generate feasibility information, preliminary effect estimates, and implementation signals rather than definitive evidence of clinical effects. The qualitative phase will explore how staff and family caregivers from intervention homes understand, implement, and appraise doll therapy in practice, including ethical concerns and cultural acceptability. Together, these components are intended to inform future larger-scale trials, non-pharmacological dementia care, medication stewardship, and implementation planning in long-term care.

### Aims and exploratory expectations

To avoid overstatement given the limited number of clusters, the study is framed as an exploratory pilot and feasibility trial. Its aims focus on feasibility, preliminary clinical and medication-stewardship signals, and implementation interpretation rather than confirmatory hypotheses about clinical effects.

*Primary registered outcome aim*. To compare class-specific mean daily medication dose for antipsychotics, anxiolytics, and antidepressants over 12 weeks between residents in nursing homes randomized to the staff-mediated doll-therapy care package plus routine nursing care and residents in homes randomized to routine nursing care alone. This aim preserves the registered primary outcome but will be interpreted as an indirect medication-stewardship signal rather than as the dominant endpoint for clinical effects.

*Main proximal clinical outcome aim*. To compare the trajectory of total NPI-12 scores at 6 and 12 weeks between the two arms (main proximal clinical outcome for interpretation).

*Secondary aims*. To compare (i) Barthel Index scores, (ii) cognitive enhancer use, and (iii) traditional Chinese medicine use over 12 weeks between the two arms, and (iv) to describe structured engagement with the doll within the intervention arm.

*Qualitative aim*. To explore how staff and family caregivers in Chinese residential dementia care understand, implement, and appraise doll therapy, including its acceptability, training needs, cultural fit, and ethical implications.

*Exploratory expectations*. Relative to routine nursing care alone, residents exposed to the doll-therapy care package may show (a) lower or stable total NPI-12 scores at 6 and 12 weeks; (b) preserved or improved Barthel Index scores; and (c) no increase in, and possibly a reduction of, class-specific daily psychotropic dose. These directional expectations are hypothesis-generating because the small number of clusters, absence of an active attention-control condition, and indirect nature of medication-related outcomes preclude confirmatory inference.

## Methods and analysis

### Study design

This study uses an explanatory sequential mixed-methods design comprising a quantitative exploratory pilot and feasibility cluster randomized controlled trial (Phase I) followed by a qualitative descriptive study (Phase II) in intervention homes. The sequential structure allows Phase II to interpret and contextualize Phase I findings, particularly with respect to implementation fidelity, acceptability, perceived benefits and harms, and ethical concerns that are difficult to capture quantitatively. Phase I is a pragmatic, parallel-group, 1:1 cluster randomized pilot trial conducted in nursing homes. Phase II is a qualitative descriptive study using semi-structured interviews with staff members and family caregivers from intervention homes. The nursing home is the unit of randomization in order to reduce contamination arising from shared staff routines, activity spaces, care culture, medication-review practices, and everyday communication among residents and staff.

The study will be conducted in six nursing homes in Zhengzhou, Henan, China. Three nursing homes will be allocated to the staff-mediated doll-therapy care package plus routine nursing care and three to routine nursing care alone. Quantitative assessments will be performed at baseline, 6 weeks, and 12 weeks. The protocol is reported in line with SPIRIT 2025 guidance for randomized trial protocols (18, 19); the structured doll-therapy care package is described in line with the Template for Intervention Description and Replication (TIDieR) checklist (20). Because only six clusters are randomized, the study should be read as an exploratory pilot and feasibility cluster trial designed to estimate recruitment, retention, fidelity, safety, acceptability, preliminary effect sizes, and parameters for a future trial. It cannot support robust clinical-effect inference or stable site-adjusted treatment-effect estimation.

Recruitment will commence after all site and ethics approvals are in place and continue until the target sample size is reached. Each enrolled participant will be followed for 12 weeks, and the overall data-collection period is anticipated to span approximately 12 to 15 months.

**Figure 1:**
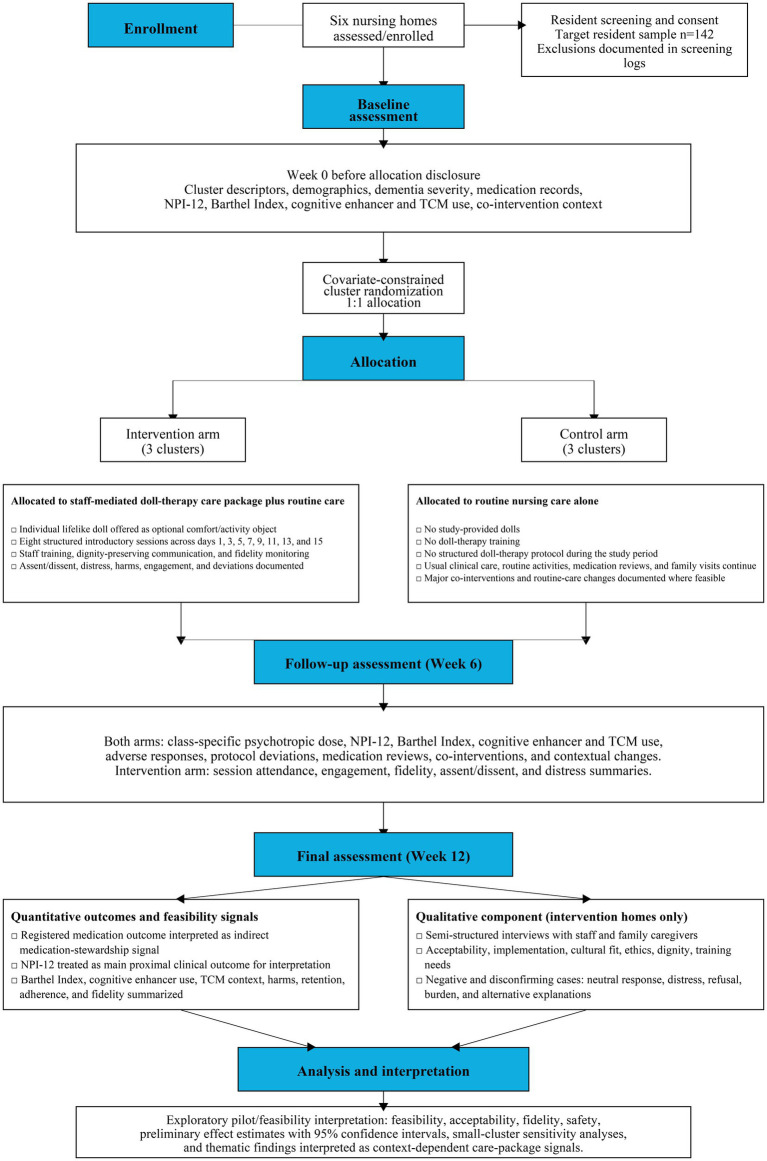
Study flow and assessment schedule for the exploratory pilot and feasibility cluster randomized trial of the staff-mediated doll-therapy care package in residential dementia care.

Patients and members of the public were not involved in the design of the protocol or the preparation of this manuscript, primarily because the research was developed within an existing residential-care service-delivery framework and timeline. Family caregiver and staff perspectives will be captured in the qualitative component, and patient and public involvement will be planned formally for a subsequent definitive trial (Figure 1).

### Setting, participants, and recruitment

Participating sites are registered nursing homes in Zhengzhou that provide dementia care and agree to comply with the study procedures. After facility-level agreement is obtained, each home will appoint a site coordinator to liaise with the research team, facilitate scheduling, support staff training, and provide access to source documents, medication records, and routine activity records.

Eligible participants must be aged 65 years or older, reside in the participating nursing home, have adequate visual capacity to recognize a doll, and have sufficient upper-limb function to hold or caress a doll safely. Adequate visual capacity is operationally defined as the ability to visually recognize a doll-sized object presented at approximately 30 cm by the screening nurse, regardless of corrected acuity; severe visual impairment documented as functional blindness in the medical record is exclusionary. Sufficient upper-limb function is operationally defined as the ability to hold an object of approximately 1.5 kg with one or both upper limbs for at least one minute in a seated position, without assistance and without pain. Both criteria will be confirmed by a trained nurse during the screening visit and documented on the screening log.

Dementia eligibility requires a documented clinical diagnosis recorded in the resident’s medical or nursing-home record by a qualified physician using DSM-5, ICD-10, ICD-11, or the most recent edition of the Chinese clinical guidelines for the diagnosis and treatment of dementia and cognitive impairment in current use at the participating sites. Where a documented dementia diagnosis exists but the underlying diagnostic criteria are not specified, the site clinician will re-confirm the diagnosis against DSM-5 criteria before enrolment. Mini-Mental State Examination, Montreal Cognitive Assessment, or equivalent baseline cognitive scores will be recorded where available (21). Dementia severity will be classified using the Clinical Dementia Rating scale, the Global Deterioration Scale, or the Functional Assessment Staging Tool where any of these is recorded in the facility’s source documentation. If no validated severity grade is available, the site clinician will assign a categorical severity (mild, moderate, or severe) based on baseline Barthel Index, NPI-12, MMSE (where available), and clinical observation, and the basis for this classification will be documented in the case report form. A legally authorized representative must be available to provide written informed consent, and resident assent will be sought whenever appropriate.

Residents will be excluded if they have acute medical instability; severe sensory or physical limitations preventing safe participation; severe communication impairment that precludes meaningful engagement or assessment of assent/dissent; urgent need for pharmacological or physical restraint; or a major psychiatric disorder unrelated to dementia that would make participation inappropriate. For the purpose of this protocol, major psychiatric disorders unrelated to dementia include documented schizophrenia spectrum disorders, bipolar disorder, current severe major depressive episode with psychotic features or high suicide risk, substance-induced psychosis, or another active psychiatric condition judged by the site clinician to require specialist treatment and to be the primary driver of behavioral symptoms. Residents with depression, anxiety, sleep disturbance, or psychotic symptoms judged to be part of dementia-related neuropsychiatric symptoms will not be excluded on this basis alone.

Potentially eligible residents will be screened from routine clinical records by trained nursing-home staff with oversight from the research team. Written informed consent will be obtained from legal guardians or authorized representatives before baseline assessment. To reduce recruitment bias, resident screening, consent, and baseline data collection will be completed before disclosure of cluster allocation whenever operationally feasible. Screening logs will document the number of residents assessed for eligibility, excluded, consented, and enrolled at each site.

### Randomization, allocation concealment, and blinding

An independent researcher who is not involved in recruitment, intervention delivery, outcome assessment, data management, or analysis will generate the 1:1 cluster allocation sequence after baseline data collection has been completed for all six participating homes. Because of the small number of clusters, covariate-constrained randomization will be used to reduce the risk of baseline imbalance on prespecified cluster-level characteristics: facility size (number of beds), dementia-care intensity (proportion of residents with dementia), staff-to-resident ratio, and aggregated baseline NPI-12 mean. The allocation algorithm will enumerate all possible 3-versus-3 cluster allocations, retain only those satisfying a predefined balance criterion on these characteristics, and draw one sequence at random from the retained set using R version 4.3 or later. The list of acceptable allocations and the chosen sequence will be archived and reported in the final trial publication. The allocation list will be held separately from site teams and released only to site coordinators after completion of baseline assessment. Baseline cluster descriptors will also be summarized before analysis to confirm balance and to document any residual imbalance for sensitivity analyses.

Because the intervention is visible in routine care, participants, direct caregivers, and staff delivering the intervention cannot be blinded. Outcome assessors will be registered nurses or research staff with prior experience in geriatric or dementia care, will not participate in intervention delivery, will be asked not to discuss group assignment with site staff, and will complete assessments in a location separate from intervention materials whenever possible. Before the trial starts, all assessors will complete a structured training program on the NPI-12 (including standardized vignettes and a reference manual) and the Barthel Index, and will demonstrate inter-rater agreement at an intraclass correlation coefficient of at least 0.75 on paired ratings of pilot vignettes; assessors who do not reach this threshold will receive additional training. Inter-rater agreement will be re-checked midway through the trial. Data files provided to the statistician will use masked group codes until the primary analysis syntax and analysis decisions have been finalized. Any accidental unblinding of assessors or statisticians will be documented, including the date, reason, and affected assessment or analysis stage.

### Intervention and comparator

The intervention is the staff-mediated doll-therapy care package plus routine nursing care. Each participant in the intervention arm will be offered an individual lifelike baby doll. The doll will be introduced as an optional comfort and activity object rather than as a real infant. Staff will avoid deceptive language that requires residents or families to accept a false belief, while also responding respectfully to residents’ spontaneous meanings and emotions. Before the intervention begins, staff will explain the purpose, potential benefits, and possible concerns to family caregivers or legally authorized representatives and will emphasize that participation is voluntary and may be paused or stopped at any time.

The estimand for this pilot trial is the effect of the doll-therapy care package as implemented in routine nursing-home care, comprising the individualized doll, staff training, structured introductory sessions, dignity-preserving communication, monitoring of assent and distress, and continued access during daily routines. The design does not isolate the doll as a stand-alone object from the additional attention, structured interaction, and relational support that accompany implementation.

Intervention deliverers must be registered nurses or certified nursing assistants with at least 6 months of dementia-care experience. All deliverers will complete an 8-h face-to-face study-specific training program covering attachment theory and person-centered care principles, the standardized activity sequence, communication skills and dignity-preserving language, assent and dissent monitoring, recognition of distress and over-attachment, safety monitoring and adverse-event documentation, infection-control procedures, and escalation routes. After training, each deliverer must pass a standardized role-play competency assessment scored by the research team against a structured checklist; deliverers who do not pass will repeat the relevant training modules before contributing to the trial.

During the introduction phase, trained deliverers will facilitate familiarization and support caregiving behaviors in a calm, familiar environment. The structured intervention comprises eight planned sessions delivered across approximately 15 days, scheduled on days 1, 3, 5, 7, 9, 11, 13, and 15. Each session is planned to last 20–30 min, adjusted to the resident’s attention, fatigue, and emotional response. The same core activity sequence will be used across intervention homes: introductory interaction with the doll, naming the doll, holding and bonding, reminiscence-oriented conversation, dressing and grooming, storytelling, music-based interaction, and shared closure activities. Sessions will be delivered individually whenever possible; small-group delivery is permitted only when consistent with the resident’s preference and the facility’s routine, and the format of every session (individual or small group) will be recorded as a process variable. After the structured phase, participants will continue to have access to the same doll within their daily routines until the 12-week follow-up.

The intervention is informed by attachment theory and person-centered care principles. It is intended to support emotional regulation, comfort, social engagement, and purposeful caregiving behavior rather than to compel any standardized response. Staff may gently invite interaction by offering the doll, asking open questions, or supporting preferred caregiving actions. Acceptable encouragement does not include insistence, repeated prompting after refusal, physical placement of the doll against the resident’s wishes, or staff statements that increase distress, guilt, or obligation.

Intervention fidelity will be monitored at three levels. First, every introductory session will be documented on a structured session checklist covering activity-sequence adherence, communication style, assent and dissent handling, and any adaptations. Second, an independent observer not involved in delivery will directly observe at least 20% of all introductory sessions at each intervention site, sampled across deliverers and across early, mid, and late phases of the introductory schedule, and will rate fidelity on the same structured checklist; inter-rater agreement between deliverers and observers will be summarized as percent agreement and Cohen’s kappa, and any systematic departure from the activity sequence will trigger a fidelity-recovery meeting. Third, follow-up access to the doll during routine care will be documented using routine activity logs and caregiver notes.

Participant engagement with the doll will be recorded as a structured process measure. Trained staff will document physical interaction, verbal interaction, caregiving behavior, duration of use, emotional response, refusal, distress, possessiveness, and any interruption or adaptation. To support reliability of engagement ratings, the research team will conduct an inter-rater agreement exercise (paired observations of at least five sessions per site) during deliverer training and again midway through the trial. These data will be used to describe intervention exposure and to support exploratory interpretation of heterogeneity in response. The activity-log template is provided as Supplementary File 1.

The structured doll-therapy activities and their conceptual basis are summarized in Table 1.

**Table 1:** Structured doll-therapy activities, planned timing, fidelity focus, and conceptual basis.

Session/planned day	Activity name	Core activity and fidelity focus	Conceptual basis
1/Day 1	Nice to Meet You	Introduce participants, staff, and caregivers; explain that the doll is an optional comfort and activity object; document assent, refusal, and baseline response.	Activity-based social engagement; person-centered introduction
2/Day 3	Thank You for Staying with Me	Introduce each participant’s individual doll; invite naming or familiarization without pressure; record acceptance and affective response.	Attachment theory; identity and belonging
3/Day 5	Love Me and Hug Me	Invite holding, touching, or comforting the doll according to preference; stop if refusal, fear, or agitation occurs.	Attachment theory; emotional regulation
4/Day 7	Listen to Me	Use open conversation or reminiscence prompts related to caregiving, family, or daily life; avoid corrective or deceptive language.	Attachment theory; reminiscence and self-identity
5/Day 9	Lovely Family	Support simple doll-care activities such as dressing or grooming; observe fine motor engagement and distress signals.	Attachment theory; purposeful activity
6/Day 11	Enjoying the Moment	Use music, storytelling, or calm shared activity with the doll; document duration and quality of interaction.	Attachment theory; positive affect and engagement
7/Day 13	Raising Babies	Simulate nurturing tasks only when welcomed by the resident; monitor possessiveness, conflict, or interference with care.	Attachment theory; caregiving role continuity
8/Day 15	Better Tomorrow	Review preferred activities, consolidate comfort routines, and plan safe continued access during routine care.	Attachment theory; continuity and closure

Sessions are scheduled on days 1, 3, 5, 7, 9, 11, 13, and 15. Each session lasts approximately 20–30 min and is adjusted to the resident’s attention, fatigue, and emotional response. Deviations from the planned schedule due to acute illness, fatigue, or staffing constraints will be documented.

The comparator is routine nursing care alone. Routine care may include assistance with eating, bathing, dressing, mobility, toileting, medication administration, health monitoring, social and recreational activities, psychological support, and communication with family members according to each facility’s usual practice. Control homes will not receive study-provided dolls, doll-therapy training, or the structured doll-therapy activity protocol during the study period. Other routine non-pharmacological activities, medication reviews, family visits, and clinical care will not be restricted because the trial is pragmatic, but major co-interventions and changes in routine care will be documented where feasible. Between-site variation in usual care will be described as contextual information and considered when interpreting the results. Because no active attention-control condition is included, observed differences will be interpreted as effects of the structured, staff-mediated doll-therapy care package, including doll use, staff training, structured interaction, relational engagement, and routine-care implementation, rather than as isolated doll-specific therapeutic effects.

### Safety monitoring and harms

Doll therapy is considered a low-risk psychosocial intervention, but potential adverse responses include distress, agitation, possessiveness, interpersonal conflict, family concern, worsening confusion, or over-attachment to the doll. Each participant in the intervention arm will be assigned an individual doll, and dolls will not be shared between residents. Dolls will be cleaned according to each facility’s infection-control procedures while preserving participants’ sense of ownership and comfort.

Resident assent will be monitored throughout the study. Refusal or dissent may be verbal, such as saying no or asking staff to remove the doll, or non-verbal, such as pushing the doll away, turning away repeatedly, crying, showing fear, increased agitation, or clear discomfort. If a resident shows refusal or distress, staff will stop the interaction for that occasion, remove or set aside the doll respectfully, provide reassurance, and record the event. Reintroduction may be attempted only when the resident is calm and willing. Two consecutive sessions with refusal or significant distress will trigger an individualized review by the site clinician; three consecutive refusals or any persistent doll-related distress will result in discontinuation of doll exposure for that participant, with the resident retained in the intention-to-treat analysis.

Adverse events will be graded as mild (transient distress resolving within the session), moderate (distress persisting beyond the session but resolving within 24 h, or non-injurious behavioral escalation), or severe (sustained distress beyond 48 h, hospitalization, injury, suspected harm to self or others, or death). All adverse events will be documented within 24 h by the site coordinator and reviewed by the principal investigator. Severe events and any unanticipated adverse events will additionally be reported to the institutional review board within 24 h of identification. Signs of persistent over-attachment, such as marked distress when separated from the doll, conflict with other residents, or interference with eating, sleep, hygiene, or clinical care, will trigger individualized review and adaptation. Safeguards to preserve dignity and autonomy include voluntary participation, proxy consent plus ongoing assent, non-deceptive introduction to families and staff, individualized stopping rules, private or respectful session settings, avoidance of infantilizing language, and documentation of adverse emotional or social reactions. Because this is a minimal-risk behavioral intervention with structured stopping rules and adverse-event review, no independent data monitoring committee is planned; this decision will be revisited if the adverse-event rate exceeds prior expectations.

### Outcomes

Outcomes are organized into a transparent hierarchy that distinguishes (i) the outcome registered in ClinicalTrials.gov, (ii) the outcome given interpretive primacy for proximal clinical response, (iii) secondary clinical outcomes, and (iv) exploratory contextual outcomes and process measures. This structure is intended to clarify what the trial can describe as feasibility-oriented, preliminary, or contextual evidence, and how each outcome should be weighted in interpretation given the exploratory pilot design. Overall conclusions will be weighted first toward feasibility, acceptability, fidelity, safety, and implementation signals; second toward NPI-12 as the validated proximal behavioral outcome; and only cautiously toward medication outcomes as indirect medication-stewardship signals.

### Registered primary outcome

The registered primary outcome (ClinicalTrials.gov NCT06506487) is class-specific mean daily medication dose for antipsychotics, anxiolytics, and antidepressants, measured at baseline, 6 weeks, and 12 weeks. Medication burden will be analyzed separately by drug class rather than as a single combined metric. These outcomes are clinically and policy-relevant because psychotropic exposure is common and potentially harmful in residential dementia care. However, they will not be interpreted as direct indicators of doll-therapy clinical effects or as the dominant basis for trial conclusions. They will instead be interpreted as indirect medication-stewardship signals because prescribing in nursing homes is clinician-led and shaped by physician practice, medication-review routines, family preferences, staffing capacity, acute illness, institutional policies, and medication-review opportunities.

Antipsychotic exposure will be standardized using chlorpromazine daily equivalents. Anxiolytic exposure will be standardized using diazepam daily equivalents for benzodiazepines and z-drugs; non-benzodiazepine, non-z-drug anxiolytics (for example, buspirone or hydroxyzine) will be quantified separately using the World Health Organization Defined Daily Dose (DDD) framework and described alongside benzodiazepine and z-drug equivalents. Antidepressant exposure will be quantified using the WHO DDD framework (22, 23). For each assessment point, the mean daily administered dose over the preceding 7 days will be calculated from medication administration records and physician orders, including scheduled and as-needed administrations where applicable. Participants not receiving a given medication class during the relevant assessment window will be assigned a dose of zero for that class. Because class-specific medication distributions may be sparse, skewed, or zero-inflated, each class will also be summarized descriptively using exposed counts and percentages, zero-exposure counts, medians and interquartile ranges, and initiation, discontinuation, and dose-reduction status where sample size permits. Psychotropic polypharmacy patterns will be reported descriptively at baseline, 6 weeks, and 12 weeks, including no exposure, monotherapy, two-class exposure, three-class exposure, and the most common class combinations. Any acute clinical events, hospital transfers, or documented medication reviews during the assessment window will be recorded where available to support cautious interpretation.

### Main proximal clinical outcome for interpretation

The main proximal clinical outcome for interpretation is the total Neuropsychiatric Inventory-12 (NPI-12) score, measured at baseline, 6 weeks, and 12 weeks, with between-group change at 6 and 12 weeks treated as the principal proximal clinical signal. The NPI-12 is a caregiver-rated instrument covering 12 neuropsychiatric domains (3). The NPI-12 was selected as the most direct, validated, behaviorally proximal measure of intervention response available in this trial. This terminology does not change the registered primary outcome; it clarifies how medication-related and behavioral outcomes will be weighted in interpretation.

### Secondary clinical outcomes

Activities of daily living will be measured using the modified Barthel Index (Chinese-language version commonly used in domestic long-term care research) scored from 0 to 100, with higher scores indicating greater independence (24, 25). Cognitive enhancer use will be examined as a secondary medication outcome and standardized using donepezil daily equivalents (26). Both outcomes will be assessed at baseline, 6 weeks, and 12 weeks.

### Exploratory contextual outcomes and process measures

Traditional Chinese medicine (TCM) use will be documented as an exploratory contextual medication outcome using a structured study-specific score that captures formula complexity, daily dosage, frequency, and duration. The TCM score has not been externally validated and will not be used for strong inferential claims; it is included to describe a locally relevant care context and to support exploratory interpretation of concurrent treatment patterns.

The scoring framework for traditional Chinese medicine use is summarized in Table 2.

**Table 2:** Study-specific exploratory scoring framework for traditional Chinese medicine use.

Scale	Scale description	Points
Complexity of formula	Based on the number of different herbs in the formula.	1 point: 1–5 herbs 2 points: 6–10 herbs 3 points: 11–15 herbs 4 points: >15 herbs
Dosage of formula	Based on the total daily dosage of the TCM formulation.	1 point: <=10 grams/day 2 points: 11–20 grams/day 3 points: >20 grams/day
Frequency of administration	Based on how often the formula is taken each day.	1 point: once daily 2 points: twice daily 3 points: three times or more daily
Duration of treatment	Based on the length of time the treatment is prescribed.	1 point: <1 month 2 points: 1–3 months 3 points: >3 months

This scoring framework was developed for the present study and has not been externally validated. It will be used as an exploratory contextual descriptor of concurrent traditional Chinese medicine use and not for strong inferential claims.

Structured engagement with the doll will be documented in the intervention arm as a process and exploratory outcome. Engagement indicators will include attendance at planned sessions, session duration, session format (individual or small group), physical interaction with the doll, verbal interaction, caregiving behaviors, positive affect, refusal, distress, interruption, and continued spontaneous use during routine care. Engagement data will be summarized descriptively and used to contextualize variability in NPI-12, Barthel Index, and medication-related outcomes.

### Participant- and cluster-level covariates

Participant-level variables will include age, sex, educational level, marital status, parental status, dementia type, dementia severity category, years since diagnosis, length of stay in the nursing home, comorbidity burden, baseline medication exposure, social interaction level, weekly participation in other therapeutic activities, and attachment-related or caregiving-history indicators (for example, prior parenthood, prior caregiving role, or doll or pet experience) where these are available from caregiver report or facility records. Cluster-level descriptors will include facility size, staffing pattern, dementia-care intensity, usual recreational activities, medication-review routines, and relevant co-interventions where available.

### Qualitative component

Phase II will use descriptive semi-structured interviews with staff members and family caregivers from intervention homes. Participants will be purposively sampled after sufficient exposure to the intervention to capture variation in professional role, facility, relationship to the resident, observed resident response, family perspective, and favorable, neutral, or unfavorable experiences. The anticipated sample is approximately 12–18 staff members and 6–12 family caregivers, with final recruitment guided by information power: sampling will continue until the data provide sufficient depth and variation to address the implementation, acceptability, and ethical questions of the study (27). Interview topics will include first impressions of doll therapy, observed resident responses, perceived benefits and harms, neutral or absent responses, refusal or distress, implementation challenges, training needs, cultural fit, ethical concerns, family communication, dignity and autonomy, and alternative explanations such as staff attention, structured interaction, or wider care-context changes.

Interviews will be conducted one-to-one in Chinese, audio-recorded with permission, transcribed verbatim, anonymized, and analyzed using Braun and Clarke’s reflexive thematic analysis framework (28). Coding will proceed iteratively. At least two members of the research team will review an initial subset of transcripts to discuss code definitions, emerging themes, and differences in interpretation. The coding framework will then be refined through team discussion and applied to subsequent transcripts, with active attention to negative and disconfirming cases, including accounts of refusal, distress, lack of benefit, family concern, staff burden, or explanations that attribute observed changes to attention or routine care rather than to the doll. Reflexive notes, coding decisions, and an audit trail will be maintained to support transparency and trustworthiness; the research team’s professional backgrounds, prior experience of dementia care, and assumptions about doll therapy will be documented at the outset and revisited as analysis proceeds, in line with reflexive qualitative practice. Reporting will follow the Consolidated Criteria for Reporting Qualitative Research (29). Because interviews are limited to intervention homes, the qualitative findings may be vulnerable to positivity bias and cannot provide a direct comparison with control-site staff experiences; this limitation will be stated explicitly in reporting.

### Sample size

No previous doll-therapy study in this setting has provided directly usable estimates for a cluster-level sample size calculation based on daily medication dose as the primary outcome. The target sample size was therefore derived pragmatically as a feasibility and planning target rather than as a definitive power calculation. The earlier participant-level G*Power calculation is retained only as a contextual planning anchor: using G*Power 3.1 (*F* tests, ANCOVA: fixed effects, main effects, and interactions; number of groups = 2; number of covariates = 1; numerator degrees of freedom = 1), assuming a medium effect size (Cohen’s *f* = 0.25), alpha = 0.05, and power = 0.80, 128 participants would be required for an individually randomized analysis of covariance including the baseline value of the outcome as a covariate. This calculation does not represent cluster-level power for the present design. Allowing for approximately 10% attrition due to acute deterioration, hospital admission, withdrawal, or death, the participant-level recruitment target was set at 142 to support feasibility estimation and preliminary signal detection.

Because the trial uses cluster randomization, the design effect was considered using the standard formula 1 + (*m* − 1) × ICC, where m is the average cluster size. With 142 participants across six nursing homes, the average cluster size is approximately 23.7. Directly comparable intracluster correlation coefficient (ICC) estimates for psychotropic medication-dose outcomes in Chinese dementia nursing homes are not available in the published literature, and empirical ICC values in long-term care vary substantially across outcomes, populations, and care settings (30–32). Sample size in cluster trials is also known to be highly sensitive to small differences in the assumed ICC, so any single value can only be treated as a planning assumption rather than as definitive (30). The most directly relevant published precedent is a UK cluster randomized trial of enhanced psychosocial care on antipsychotic use in nursing home residents with severe dementia, which used an ICC of 0.05 (33). Reported ICCs for related facility-level outcomes in older adults and healthcare cluster trials span approximately 0.005 to 0.10 depending on the outcome domain (31, 32).

To make this uncertainty transparent, we describe design effects and effective sample sizes across plausible ICC values rather than relying on a single point estimate. Under a low ICC of 0.005, the design effect is approximately 1.11 and the effective sample size is approximately 128. Under a moderate ICC of 0.02, the design effect is approximately 1.45 and the effective sample size is approximately 98. Under an ICC of 0.05, consistent with the most directly comparable nursing-home antipsychotic trial (33), the design effect is approximately 2.14 and the effective sample size is approximately 66. Under an ICC of 0.10, the design effect rises to approximately 3.27 and the effective sample size falls to approximately 43. These figures are descriptive sensitivity scenarios rather than formal power claims. They make explicit that the trial is unlikely to detect small or modest effects with confidence at any plausible ICC and reinforce the pilot and feasibility interpretation of all between-group estimates.

Because NPI-12 has been designated as the main proximal clinical outcome for interpretation, the same sample size and ICC scenarios were used to provide a directional sense of what behavioral signal might be detectable. Assuming a between-group standardized difference of approximately 0.5 standard deviations on the total NPI-12 score (i.e., approximately 5–7 points if the population standard deviation is in the 10–15 range typical of nursing-home dementia samples), the design might provide a preliminary signal under low ICC assumptions but would remain underpowered and imprecise for smaller changes or for moderate to high ICC values. NPI-12 changes will therefore be interpreted using effect estimates, 95% confidence intervals, and consistency across descriptive and cluster-level sensitivity analyses rather than dichotomous hypothesis testing.

The small number of randomized clusters is itself an important design limitation. With only three nursing homes per arm, the study is vulnerable to baseline imbalance, imprecise cluster-level effect estimates, unstable model-based standard errors, and sensitivity to site-specific practices. The trial is therefore not intended to support definitive claims about clinical effects. Instead, it will estimate recruitment, retention, adherence, fidelity, safety, acceptability, preliminary effect sizes, confidence intervals, and contextual factors needed to design a future larger cluster randomized trial, potentially with a stepped-wedge design or an active attention-control condition. Sensitivity analyses will include cluster-level summaries and randomization-based checks to make clear what magnitude and direction of difference could plausibly be observed with this design.

### Statistical analysis

All quantitative analyses will be performed in R. Baseline characteristics will be summarized using appropriate descriptive statistics. Continuous variables will be presented as means and standard deviations or medians and interquartile ranges depending on distribution, and categorical variables as counts and percentages. Baseline characteristics will be summarized at both participant and cluster levels to assess imbalance that may be important in a trial with few clusters.

The principal quantitative analysis will follow the intention-to-treat principle and use marginal repeated-measures models to account for repeated measurements within participants and clustering by nursing home. The planned primary model is a nested generalized estimating equations (GEE) model with nursing home specified as the top-level cluster and participant nested within nursing home, using an exchangeable working correlation structure. Because only six randomized clusters are available, GEE results will be treated as model-based estimates rather than as definitive inferential tests. The primary GEE variance estimate will use a bias-corrected sandwich approach suitable for small samples, prioritizing the Mancl–DeRouen correction where implementable and using Fay–Graubard or related small-sample corrections as sensitivity checks (34, 35). Analyses will be implemented in R using packages capable of modified GEE variance estimation, such as geesmv where appropriate (36). Models will include fixed effects for group, time, and the group-by-time interaction, with the interaction terms at 6 weeks and 12 weeks treated as the main estimates of between-group change over time.

The primary GEE analysis is conducted under a working missing-completely-at-random (MCAR) assumption, which is the standard requirement for consistency of GEE estimators in the absence of additional weighting. The plausibility of MCAR will be examined by comparing baseline characteristics of participants with and without complete follow-up. To address potential departures from MCAR, sensitivity analyses will use (i) multiple imputation by chained equations under a missing-at-random (MAR) assumption with auxiliary baseline variables, and (ii) inverse-probability-of-attrition-weighted GEE. Mixed-effects models will be considered as supportive analyses only, given known instability of likelihood-based variance components with fewer than approximately 10 clusters per arm.

Given the small number of randomized clusters, conventional *p* values from GEE models may be unstable. The analysis will therefore emphasize effect estimates, 95% confidence intervals, direction and consistency of change, and clinical plausibility rather than dichotomous hypothesis testing. Cluster-level change-score summaries will be prioritized as the main robustness analysis, and randomization/permutation-based checks will be conducted over the retained covariate-constrained allocation set where feasible. These checks will be interpreted descriptively because the number of possible clusters and allocations is very small. If model convergence or standard-error estimation is unstable, non-parametric descriptive comparisons and cluster-level summaries will be emphasized over model-based *p* values. This approach follows recommendations that cluster-level methods and small-sample corrections should be considered when CRTs include few clusters, while recognizing that no analytic correction can fully overcome the design limitation of six randomized clusters (37).

Prespecified covariates may include age, sex, educational level, marital status, dementia severity category, years since diagnosis, length of nursing-home stay, baseline outcome value, baseline medication exposure, social interaction level, weekly participation in other therapeutic activities, and attachment-related or caregiving-history indicators where available. Covariate adjustment will be parsimonious because of the limited number of clusters and events, and adjusted analyses will be interpreted as sensitivity analyses rather than as a way to remove all baseline imbalance. Missing data patterns will be summarized by group and time point.

Per-protocol analysis will be conducted as a sensitivity analysis among intervention participants who accept the doll and attend at least six of the eight planned introductory sessions (>=75% adherence). The 75% threshold was selected *a priori* on the basis that the dose–response relationship of doll therapy is not established and 75% adherence is a conventional cut-off used in non-pharmacological dementia-care trials to define adequate exposure. Engagement-informed exploratory analyses may describe whether greater exposure or more positive interaction with the doll is associated with more favorable changes in NPI-12, Barthel Index, or medication-related outcomes within the intervention arm. These analyses will be hypothesis-generating only.

### Data management

Quantitative data will be recorded on standardized forms by trained personnel at each nursing home and will include demographic and clinical variables, medication records, outcome measures, co-intervention information, adverse responses, and adherence and engagement documentation. Qualitative data will include audio files, transcripts, field notes, reflexive notes, and analytic memos. Paper materials will be stored in locked cabinets, and digital files will be stored in password-protected systems with access limited to authorized study personnel.

All datasets will be de-identified before analysis using unique study identifiers. Data queries, discrepancies, and protocol deviations will be documented and resolved by the study team. Access to the final analytic dataset will be restricted to the principal investigator and approved analysts. Data cleaning and analysis decisions will be documented before unmasking group labels for the primary analysis where feasible.

## Discussion

This protocol describes an exploratory pilot and feasibility mixed-methods cluster randomized trial assessing the staff-mediated doll-therapy care package in Chinese residential dementia care. The study is designed to address several clinically and publicly relevant gaps. First, evidence for doll therapy in Chinese nursing homes remains limited despite increasing dementia burden and growing reliance on institutional long-term care. Second, the study examines medication burden together with behavioral symptoms, activities of daily living, and structured engagement, providing a broader preliminary assessment than agitation outcomes alone. Third, the qualitative component is intended to clarify how staff and family caregivers interpret doll therapy, what ethical concerns arise in practice, and what implementation conditions may support or hinder acceptability.

The cluster design is appropriate because the intervention is delivered within communal care environments where contamination would be difficult to avoid if individual residents within the same home were randomized to different conditions. At the same time, the study faces important anticipated limitations. Only six nursing homes are included, which limits statistical power, precision, stability of cluster-level estimates, and robustness of GEE variance estimation even with small-sample corrections. Baseline imbalance between groups may remain despite covariate-constrained randomization. Participant and caregiver blinding is not possible because the intervention is visible. Medication changes remain clinician-led rather than researcher-directed, so variation in prescribing may reflect physician practice, medication-review routines, acute illness, family preferences, staffing capacity, or institutional policies rather than the care package alone. The absence of an active attention control also limits attribution of any observed differences specifically to the doll rather than to increased staff attention, structured interaction, or enhanced activity exposure. The present estimand is therefore the doll-therapy care package as implemented in routine care, not the isolated effect of a doll object. To partially mitigate this attributional risk, the protocol will (i) document and report between-site variation in routine non-pharmacological activities and major co-interventions, (ii) examine exposure-response patterns using the structured engagement record within the intervention arm, and (iii) use the qualitative phase to identify whether observed changes are perceived by staff and family caregivers as specifically related to the doll, to broader caregiving interaction, or to other care-context changes; nonetheless, residual confounding by attention and activity exposure cannot be excluded in this design.

For these reasons, the findings of this trial will be interpreted cautiously and within the prespecified outcome hierarchy. The study can provide feasibility information, acceptability data, intervention-fidelity information, safety observations, preliminary effect estimates, and clinical signals, but it cannot establish definitive clinical effects. The registered medication-related outcomes will be interpreted as indirect medication-stewardship signals and contextual prescribing indicators. The NPI-12 is treated as the main proximal clinical outcome for interpretation because it provides a validated, behaviorally proximal measure of intervention response. The Barthel Index and cognitive enhancer use will be reported as secondary clinical outcomes. The traditional Chinese medicine score will be treated as a contextual descriptor because it is study-specific and not externally validated. Maintaining these distinctions between feasibility outcomes, the registered medication outcome, the proximal NPI-12 clinical signal, secondary clinical outcomes, and exploratory contextual measures is essential for preventing overinterpretation, particularly in a small-cluster pragmatic pilot trial.

The ethical and cultural dimensions of doll therapy are also central to this protocol. Doll therapy may be comforting and meaningful for some residents but inappropriate or distressing for others. Implementation must therefore preserve dignity and autonomy, avoid coercion and infantilizing language, respect resident assent and dissent, and include family communication and staff training. The qualitative phase will help determine whether the intervention is acceptable in Chinese residential care, how staff and families understand its meaning, and how safeguards may need to be adapted in future studies. Because qualitative interviews are limited to staff and family caregivers from intervention homes, the analysis may overrepresent participants exposed to the intervention and may be vulnerable to positivity bias. The interview guide and analysis will therefore actively seek accounts of neutral responses, distress, refusal, family concern, staff burden, and alternative explanations for observed change, but comparative qualitative interpretation with control-site staff will remain beyond the scope of this pilot protocol.

Despite these constraints, the study has potential relevance for ageing and public health. Safer non-pharmacological approaches to dementia care are needed in residential settings where psychotropic medications remain common and staffing pressures are substantial. If doll therapy proves feasible, acceptable, safe, and associated with favorable preliminary signals when delivered with staff training, ethical safeguards, fidelity monitoring, and family communication, it may offer a practical and low-cost addition to person-centered nursing-home care in rapidly ageing settings. The results will also provide methodological information for a future adequately powered cluster randomized trial with stronger control conditions, such as a structured social-interaction attention-control arm, a larger number of randomized clusters, and more precise cluster-level inference.

### Ethics and dissemination

The study was approved by the Institutional Review Board of the School of Medicine, Sias University (approval number LL20240509). The trial was prospectively registered at ClinicalTrials.gov (NCT06506487) on July 16, 2024. Written informed consent will be obtained from legally authorized representatives of participating residents, and assent will be sought from residents whenever appropriate. Separate written informed consent will be obtained from all qualitative interview participants.

The intervention will be introduced to legal representatives and family caregivers as an optional psychosocial activity designed to support comfort and engagement. Residents will be approached respectfully, and their verbal and non-verbal assent or dissent will be monitored continuously. Participation in doll therapy will be paused or stopped when residents show refusal, persistent distress, interpersonal conflict, or over-attachment that interferes with care or wellbeing. These procedures are intended to preserve dignity, autonomy, and safety while recognizing that proxy consent alone is insufficient for an ongoing psychosocial intervention among people living with dementia.

Any important protocol amendments will be communicated to participating sites and the ethics committee as appropriate. Findings will be disseminated through peer-reviewed publication, conference presentation, and a brief lay summary for participating nursing homes and family caregivers after study completion.
