# Supplementary material for: Doll therapy in residential dementia care in China: protocol for an exploratory pilot and feasibility mixed-methods cluster randomized controlled trial
Source: Front Public Health. 2026 Jul 2;14:1842340. doi: 10.3389/fpubh.2026.1842340 (PMC13372967; doi:10.3389/fpubh.2026.1842340)
Supplement: Supplementary file 1 [file Supplementary_file_1.DOCX]

Supplementary File 1. Activity log template used to document participant engagement with doll therapy

Participant ID: ____________________ Nursing home: ____________________ Study week: __________

This template documents structured and spontaneous engagement with the assigned doll outside and during planned intervention sessions. Staff should record both positive engagement and refusal/distress so that exposure, fidelity, and safety can be interpreted transparently.

Codes: A = physical interaction, such as holding, hugging, touching, or grooming the doll. B = verbal interaction, such as talking, singing, or cooing to the doll. C = caregiving behavior, such as covering, dressing, feeding, or protecting the doll. D = positive affect or calm engagement. R = refusal/dissent. X = distress, agitation, conflict, or over-attachment requiring interruption or adaptation.

| **Date** | **Planned session?** | **Shift / time** | **Duration** | **A** | **B** | **C** | **D** | **R** | **X** | **Notes and action taken** |
| --- | --- | --- | --- | --- | --- | --- | --- | --- | --- | --- |
|  |  |  |  |  |  |  |  |  |  |  |
|  |  |  |  |  |  |  |  |  |  |  |
|  |  |  |  |  |  |  |  |  |  |  |
|  |  |  |  |  |  |  |  |  |  |  |
|  |  |  |  |  |  |  |  |  |  |  |
|  |  |  |  |  |  |  |  |  |  |  |
|  |  |  |  |  |  |  |  |  |  |  |
|  |  |  |  |  |  |  |  |  |  |  |
|  |  |  |  |  |  |  |  |  |  |  |
| Total / summary |  |  |  |  |  |  |  |  |  |  |

*Completion guidance: if R or X is recorded, describe the resident's verbal/non-verbal response, whether the doll was removed or the session stopped, who was notified, and whether reintroduction is appropriate.*
